# In Vitro Evaluation of a Gelatin Type A/PVA Hydrogel Functionalized with Roasted Green Tea (*Camellia sinensis*)

**DOI:** 10.3390/gels11110920

**Published:** 2025-11-18

**Authors:** Maria Clarisa Salazar-Nava, Rene Garcia-Contreras, Benjamin Aranda-Herrera, Gabriela Hernandez-Gomez, Carlos A. Jurado, Abdulrahman Alshabib, Patricia Alejandra Chavez-Granados

**Affiliations:** 1 Interdisciplinary Research Laboratory, Nanostructures and Biomaterials Area, National School of Higher Studies (ENES), National Autonomous University of Mexico (UNAM), Leon 37684, Mexico; srclarisa@gmail.com (M.C.S.-N.); rgarciac@enes.unam.mx (R.G.-C.); benja.aherrera@gmail.com (B.A.-H.); 2 Periodontics and Implantology Area, National School of Higher Studies (ENES), National Autonomous University of Mexico (UNAM), Leon 37684, Mexico; ghernandezg@enes.unam.mx; 3 Division of Operative Dentistry, Department of General Dentistry, College of Dentistry, The University of Tennessee Health Science Center, Memphis, TN 38103, USA; cjurado@uthsc.edu; 4 School of Dental Medicine, Ponce Health Sciences University, Ponce, PR 00732, USA; 5 Department of Restorative Dentistry, College of Dentistry, King Saud University, Riyadh 11545, Saudi Arabia

**Keywords:** *Camellia sinensis*, roasted green tea, gelatin type A, PVA, hydrogel, antimicrobial, tissue regeneration

## Abstract

Hydrogels are versatile biomaterials for controlled drug delivery and tissue regeneration due to their biocompatibility and tunable degradation. Hydrogel was synthesized with a gelatin type A/polyvinyl alcohol functionalized with aqueous extract of roasted green tea (10% *w*/*v*) and evaluated its physiobiological performance in vitro. Degradation was assessed under enzymatic (collagenase II, trypsin) and hydrolytic conditions; swelling was performed with distilled water, cytocompatibility was tested on human periodontal ligament stem cells by MTT; antibacterial activity was measured against *Streptococcus mutans*, *Staphylococcus aureus*, and *Escherichia coli*. The hydrogel showed complete hydrolytic degradation within 60 min and enzymatic degradation within 70 min, the hydrogel increased its mass by approximately 6.3 times relative weight, reached its maximum swelling in the range of 478–537%, (19% for the experimental group), while maintaining PDLSC viability (>80%). It exhibited significant antibacterial activity (inhibition: *S. aureus* 78.6%, *S. mutans* 67.4%, *E. coli* 73.2%). Importantly, in osteogenic medium, the hydrogel enhanced osteogenic differentiation of PDLSCs, evidenced by increased calcium deposition and positive Alizarin Red staining versus controls. These data position the gelatin/PVA/roasted green tea hydrogel as a bioactive, resorbable candidate for dental applications—particularly as an antimicrobial dressing and adjunct for periodontal bone regeneration material.

## 1. Introduction

In recent decades, research has focused on new alternatives to conventional pharmacological treatments due to their potential side effects and sometimes limited efficacy, as well as the need to promote tissue regeneration [1]. The main goal of these new approaches is to develop biocompatible systems capable of gradually releasing therapeutic agents. Hydrogels have been identified as materials that fulfill these desired functions [2]. Hydrogels are hydrophilic polymer networks with a three-dimensional structure capable of absorbing large amounts of water and swelling without losing their shape [3]. Key properties of hydrogels include excellent biocompatibility (due to their soft and elastic consistency) and inertness (cells generally do not adhere to their surface). They have gained more attention from bone tissue engineering (BTE) because of the possibility of designing biomaterials where the matrix is charged with molecules to induce osteogenesis [4]. Recent studies highlight the diversity of hydrogel designs tailored for specific functions. Polymeric hydrogels composed of structural materials such as sodium alginate (SA), agarose (AG), polyvinyl alcohol (PVA), and gelatin (G) have been employed in dual-network systems due to their high biocompatibility and promising potential for commercial applications [5]. Other studies have developed EDTA-inspired polydentate hydrogels, which exhibit high heavy metal adsorption capacity, positioning them as efficient and reusable adsorbents for wastewater treatment [6]. In the biomedical field, peptide-based nanogels self-assembled via metal ion coordination have shown selective antibacterial activity, providing strategies to combat multidrug-resistant infections while minimizing systemic toxicity [7]. Light-sensitive nanogels functionalized with metal ions represent an emerging class of materials capable of ultrafast light-activated responses, enabling precise spatiotemporal control in drug release for in vivo anticancer therapies [8]. Natural polymers and their combinations with various nanoparticles or natural compounds to create hydrogel systems with enhanced properties for bone regeneration [4]. These studies highlight the versatility of hydrogel-based systems and emphasize critical design parameters—such as chemical functionality, network architecture, and stimulus responsiveness—that determine their practical effectiveness in diverse applications.

Green tea (GT) is a plant of Chinese origin that has many demonstrated health benefits and is among the most popular beverages worldwide [9]. GT leaves are rich in catechins, which confer a variety of beneficial properties such as anti-inflammatory, anti-arthritic, anticancer, anti-mutagenic, antibacterial, anti-viral, anti-fungal, anti-parasitic, and hypocholesterolemia effects [10]. Given these catechin-related properties, studies have found positive effects of GT in the oral cavity, including improved periodontal health and protection against dental infections. GT has also been effective in reducing volatile sulfur compounds, thus helping prevent halitosis [11].

The use of natural extracts has increased over the past decades due to their biological properties, which are primarily attributed to the presence of phenols and flavanols [12]. These compounds should be identified to determine whether a specific biological mechanism can be associated with a particular compound. The composition and concentration of phytochemicals depend on both biotic and abiotic factors, including plant subspecies, cultivar, geographical origin of the cultivar, environmental conditions, and harvesting season [13]. Such variability can significantly influence the reproducibility and biological performance of biomaterials incorporating plant-derived compounds. In this study, a commercial roasted green tea (RGT) of Japanese origin was used. The phytochemical composition of the aqueous extract was previously reported, showing it is rich in gallic acid, (–)–Gallocatechin, (–)–Epigallocatechin, (–)–Catechin, Caffeine, (–)–Epigallocatechin gallate, and (–)–Epicatechin. In the same study, the extract was quantified by HPLC [14]. This data provides a reliable basis for its use in hydrogel design, allowing for a more consistent interpretation of its functional contribution.

These attributes make RGT a compelling functional additive for biomedical materials. In this work, the objective was to incorporate aqueous extract of roasted green tea (AERGT) into a gelatin/Polyvinyl alcohol hydrogel (G/AERGT/PVA) to evaluate whether the resulting composite hydrogel could provide antimicrobial activity while maintaining biocompatibility and suitable degradation properties for potential clinical use. The hypothesis here was related to that loading the hydrogel with an AERGT rich in catechins (C) would (i) produce measurable inhibition of representative oral pathogens (e.g., *Streptococcus mutans*, *Staphylococcus aureus*, and *Escherichia coli*), (ii) preserve cytocompatibility with human periodontal ligament stem cells (PDLSCs), and (iii) yield a short, controllable degradation profile (on the order of ~1 h) under hydrolytic and enzymatic conditions compatible with short-term intraoral applications.

## 2. Results and Discussion

### 2.1. PDLSCs Characterization and Cytotoxicity of Hydrogels

**Figure 1:**
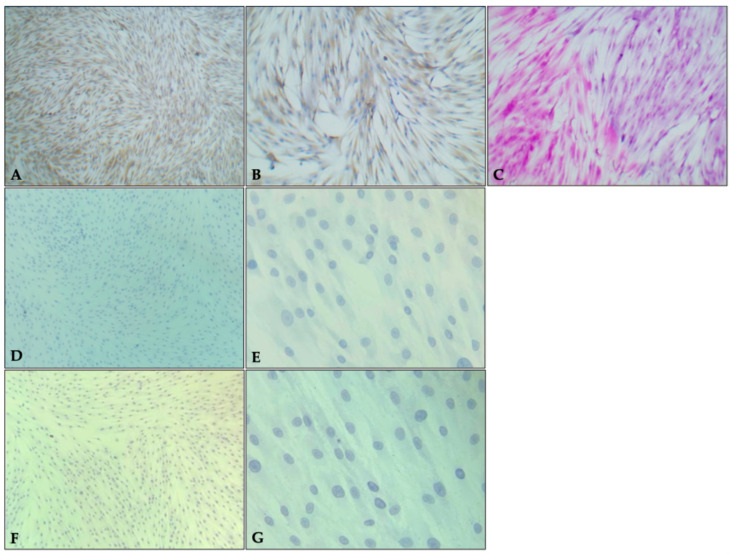
Characterization of primary PDLSCs by immunohistochemistry. Representative photomicrographs showing strong immunopositivity for vimentin (+++) ((**A**) = 10×, (**B**) = 40×), intense positivity with Masson’s trichrome staining indicating connective tissue morphology ((**C**) = 40×), and negative immunoreactivity for CD34 ((**D**) = 10×, (**E**) = 40×) and CD56 ((**F**) = 10×, (**G**) = 40×).

Primary PDLSCs cultures showed positive staining for Masson’s trichrome staining revealed cells with predominantly oval and some polygonal morphologies, displaying basophilic cytoplasm, evident nuclei and nucleoli, and condensed chromatin organized within a fibrous stromal network of connective tissue with strong positivity. Immunohistochemical analysis confirmed a strong (+++) cytoplasmic signal for vimentin, while hematopoietic/endothelial markers CD34 and CD56 were negative (Figure 1).

**Figure 2:**
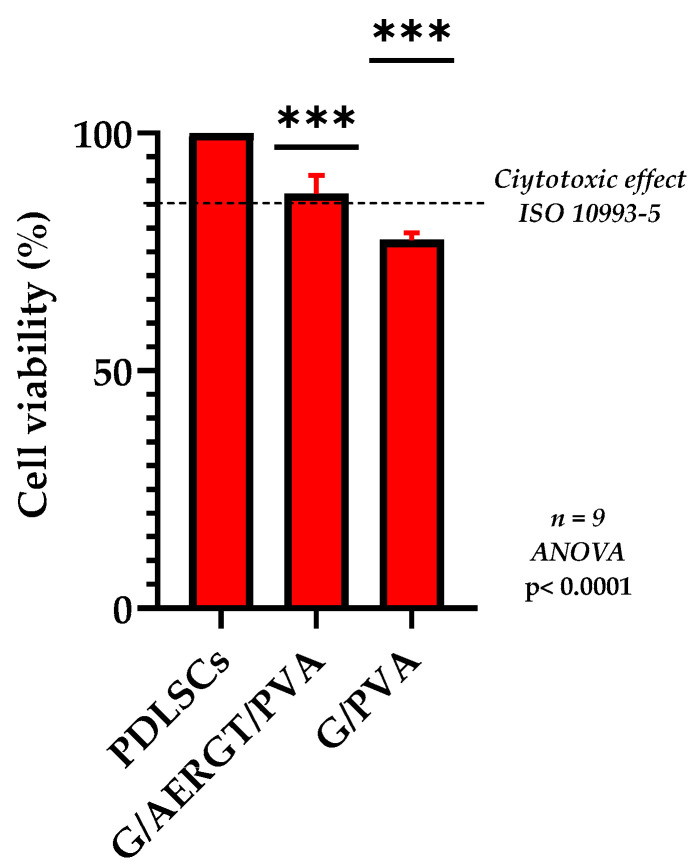
Cell viability of PDLSCs after 24 h incubation with hydrogels in the MTT assay. The bar graph shows the percentage of viable cells in the presence of G/AERGT/PVA hydrogel and the control hydrogel (G/PVA), relative to untreated control cells (100%). Data means SD (n = 9). The experimental hydrogel (with roasted green tea) maintained high cell viability (~87%) comparable to the control hydrogel (~77%), indicating low cytotoxicity of both formulations. The dashed line represents the threshold for cytotoxic effect based on ISO 10993-5 [15]. *** *p* < 0.001, indicating a statistically significant difference between groups.

After 24 h of exposure to the hydrogels, the PDLSCs viability in the MTT assay remained high for both hydrogel groups. Figure 2 summarizes the cell viability results. The PDLSCs cultured with the roasted green-tea-loaded hydrogel (G/AERGT/PVA) exhibited about 87.3% viability relative to untreated control cells, whereas those with the non-functionalized hydrogel (G/PVA) showed approximately 77.5% viability. There was no significant cytotoxic effect observed for either hydrogel, as cell viability stayed above 70% in both cases. While the experimental hydrogel showed slightly higher cell viability than the plain hydrogel, neither caused appreciable cell death or morphological signs of cytotoxicity. These results indicate that the incorporation of the AERGT did not induce cytotoxic effects on human periodontal ligament stem cells and that the developed hydrogels are cytocompatibility.

In the cytotoxicity assays, the G/AERGT/PVA hydrogel showed no cytotoxic effect on PDLSCs, like the control hydrogel without RGT, and neither did hydrogel promote significant cell proliferation. Both maintained cell viability well above the cytotoxicity threshold, with the RGT hydrogel performing slightly better.

### 2.2. Enzymatic and Hydrolytic Degradation Tests

**Figure 3:**
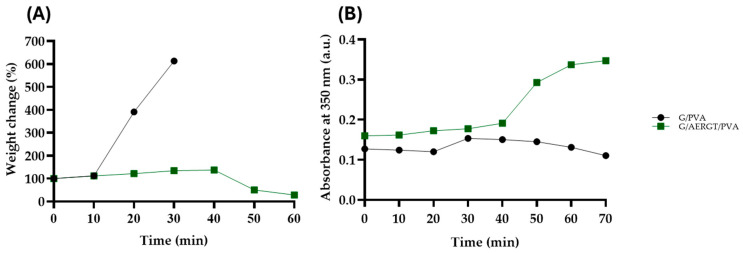
Hydrolytic change in G/AERGT/PVA hydrogels in PBS at 37 °C. (**A**) Weight change (%) of hydrogels over time. Both samples exhibited an initial increase due to water absorption and matrix hydration during the first 30–40 min, followed by gradual weight loss associated with dissolution and hydrolytic degradation. The G/PVA hydrogel reached a higher swelling ratio (~537%) (0.20 ± 0.25 ANOVA *p* = 0.0003) whereas G/AERGT/PVA showed a limited increase (~19) (0.05 ± 0.12 ANOVA *p* = 0.003). (**B**) Absorbance of the PBS supernatant at 350 nm as a function of time, indicating the release of gelatin fragments, tea polyphenols, and other soluble components. The G/AERGT/PVA (0.230 ± 0.081) hydrogel exhibited higher absorbance values after 40 min, suggesting greater release of degradation products compared with G/PVA (0.132 ± 0.016 at 70 min, ANOVA, *p* < 0.0001, n = 9).

The weight change in the G/PVA and G/AERGT/PVA hydrogels in PBS at 37 °C is presented in Figure 3. During the first 30–40 min, both hydrogels (Figure 3A) exhibited an initial weight increase, which can be attributed to water absorption and hydration of the polymeric matrix. After this hydration phase, gradual weight loss was observed, corresponding to partial dissolution. Since these data were obtained from hydrated samples, the observed changes represent the combined effects of swelling, dissolution, and degradation, rather than pure swelling alone. It should also be noted that hydrolytic degradation may begin concurrently with the swelling phase, even if not yet reflected in the weight profile.

The G/PVA hydrogel showed a higher hydration capacity, reaching approximately a 5.3-fold increase (~537%), while the G/AERGT/PVA hydrogel exhibited a reduced hydration (~19% increase). This difference may result from hydrogen bonding and hydrophobic interactions between polyphenols in the roasted green tea extract and the functional groups of gelatins and PVA, which limit the number of hydrophilic sites available for water binding [16,17,18]. Such interactions modulate the hydrogel’s physicochemical properties and influence its water uptake and degradation behavior under physiological conditions. As shown in Figure 3B, the absorbance of the PBS solutions at 350 nm increased overtime for both hydrogels, reflecting the progressive release of soluble components such as gelatin and tea polyphenols as the network structure underwent hydrolytic degradation. The G/AERGT/PVA hydrogel exhibited slightly higher absorbance values than the G/PVA control, particularly after 40 min, likely due to the contribution of roasted green tea extract, which naturally absorbs in the UV range. Although the weight variation data provides insight into swelling and mass loss behavior, they do not directly quantify the extent of degradation. Therefore, the absorbance profile was considered a more sensitive indicator of the hydrolytic breakdown and release process occurring during the first 60 min.

**Figure 4:**
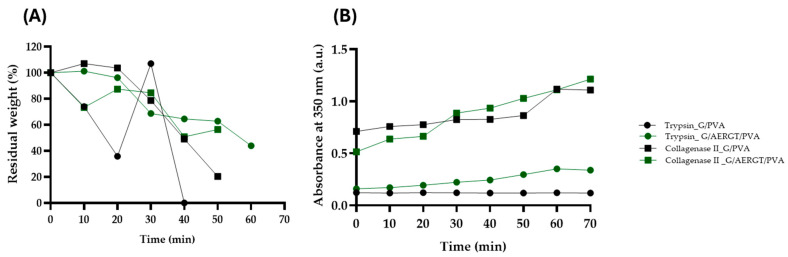
Weight variation and absorbance changes in G/PVA and G/AERGT/PVA hydrogels under enzymatic conditions (collagenase II and trypsin, 37 °C). (**A**) Residual weight (%) of G/PVA and G/AERGT/PVA hydrogels as a function of time. Under collagenase II, G/AERGT/PVA fluctuated slightly but remained intact until ~50 min, collapsing by 70 min (remaining weight 0.05 ± 0.01), whereas G/PVA steadily lost weight and collapsed by ~50 min (0.06 ± 0.01). Under trypsin, G/AERGT/PVA (0.05 ± 0.01) showed continuous weight loss until collapsing at ~60 min, while G/PVA (0.04 ± 0.01) displayed irregular weight variations before collapsing around 50 min. By 70 min, no intact hydrogel remained under any enzymatic condition (ANOVA, *p* < 0.0001, n = 9). (**B**) Absorbance at 350 nm (a.u.) of the surrounding medium overtime. Solid squares represent collagenase II exposure, and solid circles represent trypsin exposure. Both G/PVA and G/AERGT/PVA hydrogels showed nearly overlapping absorbance curves under collagenase II (~0.87 at 70 min). In trypsin, G/AERGT/PVA exhibited an increasing absorbance (0.247 ± 0.074 at 60 min), whereas G/PVA showed a slight decrease (0.12 ± 0.002 at 60 min), suggesting different interactions with trypsin.

Enzymatic degradation was assessed using two proteolytic enzymes: collagenase type II, which simulates collagen-degrading conditions, and trypsin, a broad-spectrum protease. The results are presented in Figure 4A, B.

Residual weight (Figure 4A) provided complementary insight into hydrogel stability. In the presence of collagenase II, both G/PVA and G/AERGT/PVA hydrogels gradually lost weight within the first 60 min and were fully degraded after approximately 70 min. When exposed to trypsin, the G/AERGT/PVA hydrogel exhibited a more uniform pattern of weight loss, collapsing after ~60 min, whereas the G/PVA hydrogel showed irregular fluctuations—likely due to partial re-adsorption of fragments—before collapsing around 50 min. Complete enzymatic degradation, characterized by the loss of structural integrity, thus occurred slightly faster in trypsin (50–60 min) than in collagenase (≈70 min).

In the presence of collagenase II, both hydrogels showed a progressive increase in absorbance at 350 nm over 70 min (Figure 4B), indicating continuous breakdown of the gelatin network. The absorbance curves for G/PVA and G/AERGT/PVA were almost identical, reaching ~0.874 at 70 min, suggesting that the roasted green tea (RGT) extract did not inhibit the enzymatic activity of collagenase. This indicates that both hydrogels release gelatin fragments at comparable rates under collagenolytic conditions.

In contrast, under tryptic degradation, clear differences were observed: the G/AERGT/PVA hydrogel displayed a steady increase in absorbance (up to ~0.247 at 60 min), while the G/PVA hydrogel showed a slight decrease (~0.120 at 60 min). This behavior suggests that components of the RGT extract (e.g., polyphenols) may influence trypsin-mediated proteolysis, possibly by stabilizing the hydrogel matrix or contributing to UV absorbance at 350 nm.

These results demonstrate that hydrogel is biodegradable under enzymatic conditions, and the addition of AERGT does not significantly alter the overall degradation time frame. Importantly, the degradation times on the order of 1 h suggest that in a biological environment (with enzymes present), the hydrogel would resorb relatively quickly. This could be advantageous for short-term applications such as post-surgical wound dressings or drug delivery depots, but it may necessitate cross-linking or further modification for longer-term implants,

Our degradation findings align with previous studies of similar hydrogels. For instance, Echeverri et al. reported that PVA hydrogels at 7.5% concentration have high stability at elevated temperatures and across pH ranges which supports our use of 7.5% PVA to impart thermal and mechanical stability to the hydrogel [19]. Laya et al. found that semi-interpenetrating networks of polyacrylamide with PVA showed significantly greater water absorption capacity, stiffness, and strength compared to hydrogels without PVA [20]. This suggests that the inclusion of PVA in our formulation would likely improve the structural integrity of the hydrogel, allowing it to maintain form during the initial swelling phase. Osorio et al. demonstrated that increasing the proportion of gelatin in composite hydrogels (with chitosan and PEG) led to enhanced resistance and swelling Capacity [21]. In our system, gelatin is the primary polymer, and its relatively high content (20% w/v) may similarly contribute to the mechanical robustness of the hydrogel and its interaction with enzymes. Moreover, a study by Gorgieva and Kokol on chitosan–gelatin hydrogels showed that these hydrogels-maintained stability in the presence of collagenase and lysozyme, especially after undergoing a lyophilization process that improved their structural Properties [22]. Although we did not lyophilize our hydrogels, their rapid yet controlled degradation in enzymatic conditions indicates an adequate stability that could potentially be further tuned by cross-linking or drying methods if needed. Overall, the degradation behavior observed—rapid in pure buffer and slightly moderated in enzymatic conditions—suggests that the hydrogel could be suitable for applications requiring quick resorption. The hydrolytic (PBS) and enzymatic (trypsin and collagenase) degradation tests indicated that both the green tea hydrogel and the control hydrogel have an adequate degradation time (on the order of 1 h or less). In PBS and trypsin, the two formulations behaved very similarly, while in collagenase the presence of RGT did not adversely affect stability. The absorbance data suggested that collagenase induces more rapid breakdown (higher solubilization) than PBS or trypsin alone, but overall, the hydrogels showed comparable degradation under all conditions tested. This rapid yet controlled degradability is advantageous for intraoral use, where materials should dissolve or be easily removed within a reasonable time frame.

For longer-term applications, additional cross-linking might be necessary to slow the degradation.

### 2.3. Antibacterial Activity Against Oral Pathogens

**Figure 5:**
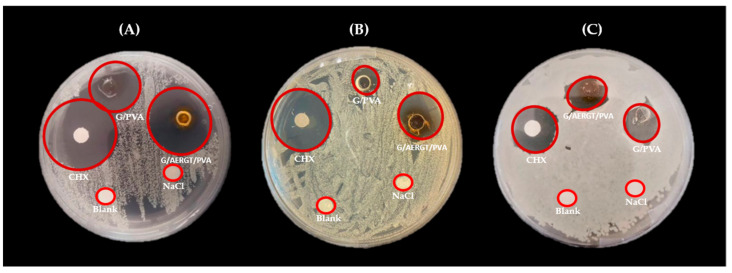
Results of the agar diffusion assays showing inhibition halos against (**A**) *S. mutans*, (**B**) *S. aureus* and (**C**) *E. coli*. Each plate contained: a G/AERGT/PVA hydrogel, a G/PVA hydrogel, a disk with 0.2% chlorhexidine (CHX, positive control), a disk with NaCl (negative control), and a blank disk. The images show that CHX produced the largest clear zones of inhibition for all bacteria. The G/PVA hydrogel and G/AERGT/PVA exhibited noticeable inhibition halos (especially for *S. aureus* and *S. mutans*), larger than those of the G/PVA hydrogel. The G/PVA hydrogel had the smallest halos, indicating minimal intrinsic antibacterial activity. Overall, *S. mutans* were the most susceptible to all treatments (largest halos), *E. coli* showed the smallest halos, and *S. aureus* was intermediate.

The hydrogel functionalized with AERGT exhibited notable antibacterial effects against the tested oral pathogens (*S. mutans*, *S. aureus*, and *E. coli*). In the agar diffusion assay, clear inhibition zones were observed around the G/AERGT/PVA hydrogel disks for all three bacteria, whereas the G/PVA hydrogel (without roasted green tea) produced only minimal zones. Figure 5 shows representative images of the inhibition halos on Mueller Hinton agar. Against *S. mutans* (Figure 5A), was somewhat more sensitive: the G/AERGT/PVA hydrogel’s halo (~8.5 mm) was nearly as large as that of the positive control (0.2% CHX disk, ~9.1 mm), and even the G/PVA hydrogel showed a measurable halo (~8.5 mm) for *S. mutans*. *S. aureus* (Figure 5B) the G/AERGT/PVA hydrogel produced a discernible inhibition halo (mean diameter ~7.3 mm), whereas the plain G/PVA hydrogel had a much smaller halo (~4–5 mm) was somewhat more sensitive: the G/AERGT/PVA hydrogel’s halo (~8.5 mm) was nearly as large as that of the positive control (0.2% CHX disk, ~9.1 mm), and even the G/PVA hydrogel showed a measurable halo (~8.5 mm) for *S. mutans*. For *E. coli* (Figure 5C), both hydrogels produced similar inhibition zones (~7.3 mm for G/AERGT/PVA and ~6.6 mm for G/PVA), which were smaller than the halo from the CHX control (~8.3 mm). Table 1 quantifies the results of the diffusion assays, listing the inhibition zone diameters for each trial and the averages. The positive control (CHX 0.2%) yielded the largest inhibition zones for all bacteria, as expected (around 9–10 mm for *S. aureus* and *S. mutans*, slightly lower for *E. coli*). The G/AERGT/PVA (experimental hydrogel) showed moderate inhibition zones: about 7.25 mm for *S. aureus* and *E. coli*, and 8.50 mm for *S. mutans*. The G/PVA hydrogel (negative control) had minimal effect on *S. aureus* (4.33 mm mean halo) and *E. coli* (~6.58 mm, which might partly be the disk size itself), but interestingly showed some inhibition of *S. mutans* (~4.75 mm). *S. mutans* may have been slightly inhibited even by the plain G/PVA hydrogel, possibly due to minor leachable or the inherent mild acidity of gelatin. Nonetheless, the addition of AERGT clearly enhanced antibacterial performance, especially against *S. aureus* and *S. mutans*.

As shown in Table 1, *S. aureus* and *E. coli* had similar responses: the RGT hydrogel achieved ~7.3 mm inhibition zones for both, whereas *S. mutans* had a slightly larger inhibition zone (~8.5 mm) with the roasted green tea hydrogel. This suggests that *S. mutans* might be particularly sensitive to components of the AERGT (or conversely, that the plain hydrogel had an unexpected mild effect on *S. mutans*, making the relative increase smaller). Although the inhibition zones appeared slightly different, statistical analysis using the Kruskal–Wallis test followed by Mann–Whitney multiple comparisons revealed no significant differences among groups (*p* > 0.05). This indicates that the agar diffusion assay provides only a qualitative indication of growth inhibition (presence or absence of bacterial death), whereas the broth microdilution method offers a more sensitive and quantitative evaluation, allowing clearer detection of antimicrobial differences.

**Figure 6:**
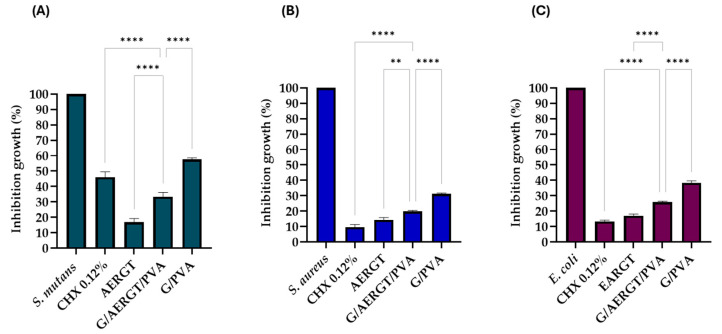
Microdilution assay results for (**A**) *S. mutans*, (**B**) *S. aureus*, and (**C**) *E. coli.* Bars represent the mean percentage of growth inhibition (relative to untreated control = 0% inhibition, 100% growth). For *S. mutans*, G/AERGT/PVA exhibited the highest inhibition (67.43 ± 2.8%), AERGT (83.16 ± 1.13), CHX 0.2% (53.92 ± 3.4%) and the G/PVA (43.02 ± 1.13%). For *S. aureus*, G/AERGT/PVA reached 78.57 ± 0.66% inhibition, compared to 90.02 ± 1.75% for CHX, AERGT 85.75 ± 1.6% and 68.31 ± 0.56% for the G/PVA hydrogel. For *E. coli*, inhibition levels were 73.16 ± 0.62% for G/AERGT/PVA, AERGT 82.93 ± 1.15%, 87.15 ± 0.88% for CHX, and 60.86 ± 1.2% G/PVA hydrogel. ** *p* < 0.01, **** *p* < 0.0001, according to two-way ANOVA followed by post hoc analysis. These significance levels indicate the probability that the observed differences occurred by chance. A higher number of asterisks corresponds to a lower probability of random variation, and therefore a stronger statistical difference between groups.

To further quantify antibacterial efficacy, we performed the microdilution MTT assay, which measures metabolic activity of bacteria in broth culture after exposure to the hydrogels. The results, shown in Figure 6, expressed as percentage inhibition of bacterial growth compared to untreated control (100% growth). For *S. mutans* (Figure 6A), the G/AERGT/PVA hydrogel achieved an inhibition of 67.43 ± 2.50%, which was notably higher than the inhibition by the positive control CHX at 0.2% (53.92 ± 2.51%). The G/PVA produced ~43.0% inhibition. Thus, against *S. mutans*, the RGT hydrogel not only outperformed the plain hydrogel but even exceeded the CHX control in this assay. For *S. aureus* (Figure 6B), the G/AERGT/PVA hydrogel inhibited 78.57 ± 0.77% of bacterial growth, whereas the 0.2% CHX control showed about 90.02 ± 1.75% inhibition. The plain hydrogel caused 68.31 ± 0.96% inhibition. So, the RGT hydrogel was effective against *S. aureus*, though chlorhexidine was superior, as expected. Against *E. coli* (Figure 6C), the G/AERGT/PVA hydrogel resulted in 73.16 ± 1.61% inhibition, compared to 87.15 ± 0.60% for CHX and 60.86 ± 0.90% for the plain hydrogel. These microdilution results corroborate the diffusion assay findings, where the hydrogel functionalized with AERGT significantly enhances antibacterial activity relative to the non-functionalized hydrogel. In particular, the effect on *S. mutans* is remarkable, suggesting AERGT is highly potent against this organism.

It is worth noting that the two antibacterial assays yielded some differences in relative performance. In the diffusion assay on solid media, CHX produced larger inhibition zones than hydrogels for all bacteria, whereas in the broth (MTT) assay, the RGT hydrogel matched or surpassed chlorhexidine for *S. mutans*. These differences could be due to the modes of action and diffusion of the agents: chlorhexidine is a diffusive small-molecule antiseptic that rapidly kills bacteria in proximity, while the RGT extract contains polyphenolic compounds that may act differently.

Additionally, the plain hydrogel showing any significant inhibition (*p* > 0.05) in the diffusion test for *S. mutans* might indicate some slight acidity or nutrient adsorption affecting bacterial growth on solid media; however, in the broth culture, the plain hydrogel did show an inhibitory effect (~43–68% depending on organism), which could be due to it absorbing some bacteria or releasing traces of chemicals (e.g., residual acetic acid from gelatin or low MW compounds from gelatin/PVA). Regardless, the presence of the AERGT clearly enhanced antimicrobial efficacy. The antimicrobial effect of the AERGT can also be observed independently (Figure 6) causing greater inhibition in bacterial growth. In the hydrogel system, however, the effect appears lower compared to the extract alone. This can be explained by the fact that the hydrogel matrix may limit direct contact between the bacteria and the active compounds, or slow down their release, producing a more controlled but delayed exposure; however, the physics of these mechanical interactions in different cell culture conditions are poorly understood [23]. Indeed, degradation studies show that significant release of hydrogel components, including tea polyphenols, occurs around 60 min. Therefore, the lower immediate effect observed in the hydrogel does not indicate lack of activity but rather a sustained and gradual release profile of the antimicrobial agents, consistent with the hydrogel’s physical properties.

Green tea’s antimicrobial properties are largely attributed to its polyphenolic constituents, particularly catechins (such as epigallocatechin-3-gallate, EGCG) [24,25]. These compounds can damage bacterial cell membranes and interfere with microbial metabolic enzymes. *S. mutans* have been identified as especially sensitive to tea polyphenols [26]. Kawarai et al. reported that green tea extracts (as well as Assam tea extracts) inhibit *S. mutans* biofilm formation by targeting bacterial glucosyltransferases—enzymes that synthesize extracellular glucans for biofilm matrix [27]. By inhibiting glucosyl transferase B, C, and D, catechins in GT prevent *S. mutans* from effectively forming biofilm and aggregating, thereby reducing its cariogenic potential [28]. This mechanism may explain why our RGT a notably strong effect on *S. mutans* viability.

In diffusion assays, the control hydrogels (G/PVA) produced small but measurable inhibition zones, suggesting a mild intrinsic antibacterial effect. This effect could be attributed to several factors: gelatin may release low levels of peptides derived from partial hydrolysis, which have been described to exhibit mild antibacterial activity in some contexts [18]; on the other hand, PVA is highly hydrophilic, and in combination with gelatin it may enhance the diffusion of such soluble components, creating a transient inhibitory effect [29]. This behavior aligns with the physicochemical interactions observed during the hydrolytic degradation of the G/PVA matrix, where rapid hydration followed by controlled dissolution favors the initial release of low-molecular-weight gelatin fragments and residual compounds. Controlled-release systems (e.g., hydrogels or polymer films loaded with tea polyphenols or plant extracts) commonly display anomalous or diffusion-controlled release kinetics, which can blunt halo size despite measurable inhibition in broth microdilution [30]. In contrast, the G/AERGT/PVA hydrogels exhibited markedly larger inhibition zones, consistent with the presence of active polyphenolic compounds from the roasted green tea extract. Therefore, while the base hydrogel displays only mild inherent activity, the pronounced inhibition observed in the AERGT-containing system confirms that the primary antibacterial effect originates from the incorporated extract, as supported by the microdilution assay results.

### 2.4. Evaluation of the Hydrogel in Bone Differentiation Processes

**Figure 7:**
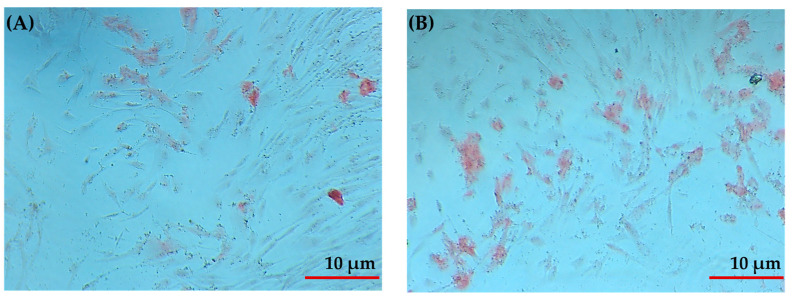
Alizarin Red staining of PDLSCs cultured in hydrogels. (**A**) Hydrogel without roasted green tea shows fewer calcium deposits, indicating lower osteogenic differentiation at 20×. (**B**) Hydrogel with RGT demonstrates increased calcium deposits, indicating enhanced osteogenic differentiation at 20×.

After obtaining positive results from the cell viability assay, the study progressed to the next phase by evaluating the hydrogel’s ability to induce osteogenic differentiation in PDLSCs (Periodontal ligament (PDL) stem-like cells). Osteogenic differentiation was assessed by detecting calcium deposits using Alizarin Red staining. The G/AERGT/PVA hydrogel showed a markedly higher level of mineralized matrix formation compared to control cultures without hydrogel. The PDLSCs cultured in the presence of the G/AERGT/PVA hydrogel produced significantly more calcium deposits, indicating enhanced osteogenic differentiation. Alizarin Red staining confirmed that the hydrogel environment effectively promoted mineralization, highlighting its potential to stimulate PDLSCs toward an osteoblastic lineage for bone tissue formation (Figure 7).

The evaluation of the G/AERGT/PVA hydrogels on PDLSCs also demonstrated a non-toxic effect. While incorporating bioactive extracts into hydrogels can endow them with antimicrobial and regenerative properties, it is crucial that these biomaterials remain safe for human cells. By integrating the bioactive compounds into a hydrogel, a localized and sustained release of the compounds directly at the injury site can be achieved, making this a highly promising strategy for targeted therapeutic applications. Chen et al. (2021) reported that base hydrogels (ODex/HA–ADH and ODex/HA–ADH/HACC) supported high viability and proliferation of L929 fibroblasts (over 90% viability), whereas incorporating silver nanoparticles into the same hydrogels (forming an Ag@ODex/HA–ADH/HACC composite) reduced cell viability to about 76%, accompanied by morphological changes in the cells [31]. This moderate cytotoxic effect was attributed to the presence of silver at higher doses, underscoring the importance of determining the optimal therapeutic dose of any incorporated agent to ensure biocompatibility. Our results confirm that the G/AERGT/PVA hydrogel can be safely used without harming healthy cells. A primary goal of this hydrogel formulation is to induce the differentiation of resident progenitor PDLSCs into an osteoblastic lineage, which is crucial for regenerating bone in periodontal defects. This strategy for bone regeneration highlights the hydrogel’s ability to actively support bone cell proliferation and guide osteogenic differentiation, while also fostering seamless integration with surrounding tissues. By combining both bioactivity and structural compatibility, the G/AERGT/PVA hydrogel emerges as a promising candidate for applications in tissue engineering and regenerative medicine.

Hydrogels are highly valued in tissue engineering due to their excellent biocompatibility and their ability to provide a supportive, three-dimensional environment for cell growth and differentiation. In this study, the G/EARGT/PVA hydrogel created a conducive microenvironment that promoted the differentiation of PDLSCs into the osteogenic lineage, as evidenced by the increased calcium deposition in the osteogenic differentiation assays. Hydrogels not only offer a scaffold that mimics the extracellular matrix but also features bioactive sites that can be functionalized for specific purposes, such as promoting tissue regeneration or delivering growth factors. Moreover, incorporating therapeutic compounds into a hydrogel allows for localized drug delivery at the site of injury, preventing the active agents from diffusing into the systemic circulation. Many hydrogels have been formulated using one or more polymers and can be loaded with various bioactive elements (such as drugs, nanoparticles, or growth factors) to enhance their functionality. For example: (i) the conjugation of chitosan/rosuvastatin nanoparticles into a sodium alginate/PVA hydrogel (forming a composite delivery system) showed high cell viability in human fibroblast cultures [32]. This indicates that such a multi-polymer hydrogel can be used for controlled drug delivery without cytotoxic effects; (ii) hydrogels based on hyaluronic acid have been widely used in bone regeneration, particularly in dental and craniofacial applications. Hyaluronic-acid hydrogels are biocompatible and can support bone healing in periodontal defects and jawbone augmentation procedures [33], (iii) hybrid “double-network” hydrogels have been designed to improve mechanical properties and osteointegration [34]. For instance, Fang Guo et al. (2020) developed a polyacrylic acid–alginate (PAA–Alg) interpenetrating network hydrogel enriched with calcium polyphosphate, which exhibited enhanced mechanical strength and improved osseointegration in bone regeneration models [35].

Beyond providing a scaffold for cells, it is important that a biomaterial used for bone regeneration also helps manage the local biological environment. Natural bioactive extracts, such as catechins from RGT, can contribute additional benefits to the regenerative material. In essence, the incorporation of such natural extracts into the hydrogel means that it not only directs stem cells toward bone formation but also creates a healthier local environment for regeneration (for example, green tea polyphenols like EGCG are known to reduce gingival inflammation and inhibit the growth of periodontal pathogens in the context of periodontitis.

Recent studies have explored the use of natural polyphenols such as EGCG (a dominant catechin in green tea) in tissue engineering to enhance bone regeneration. EGCG has been shown to bolster osteogenic differentiation of mesenchymal stem cells by increasing the expression of key markers like BMP–2, Runx2, ALP, osteonectin, and osteocalcin, and by mitigating oxidative stress and inflammation through antioxidant pathways [36].

In our study, the G/AERGT/PVA hydrogel proved capable of inducing robust osteogenic differentiation in PDLSCs, as confirmed by the pronounced mineralization in vitro. This finding underscores the promise of hydrogels functionalized with natural plant extracts as therapeutic tools in regenerative medicine, particularly for bone regeneration in periodontal contexts, where encouraging local stem cells to rebuild lost alveolar bones is a key goal [37]. The ability to promote mineralized tissue formation within a cell–populated hydrogel suggests that such biomaterials could be applied to periodontal bone defects to stimulate repair and enhance the integration of new bone with existing structures.

The pro–osteogenic effects of our extract-loaded hydrogel appear dose-dependent and involve key mechano-transduction pathways. Prior research in periodontal-ligament-derived cells has implicated the RhoA–TAZ (Ras Homolog Family Member A–Transcriptional coactivator with PDZ–binding motif) axis in mediating osteogenic differentiation. RhoA enhances cytoskeletal tension and focal adhesions, which in turn promote RhoA–TAZ–mediated osteogenesis. TAZ—an effector of the Hippo pathway—is crucial for osteoblast lineage commitment and sustaining bone regeneration, as its suppression markedly attenuates differentiation. Furthermore, in the context of periodontal ligament stem cells, TAZ enhances osteogenesis even under inflammatory conditions, such as LPS exposure. Together, these data suggest that our G/AERT/PVA hydrogel may support bone regeneration by engaging the RhoA–TAZ pathway, thereby transducing mechanical cues into biochemical signals that promote osteogenic gene activation—a sophisticated hybrid strategy combining matrix support with bioactive signaling in regenerative medicine.

### 2.5. Potential Clinical Implications

The combination of results from cytotoxicity, degradation, and antimicrobial tests suggests that the G/AERGT/PVA hydrogel could be a viable material for certain clinical uses, especially in dentistry. The hydrogel did not induce cytotoxicity in PDLSCs and thus is likely biocompatible with oral tissue cells. It degraded within about an hour in physiological conditions, which means it would not persist long-term—this could be ideal for a resorbable dressing or a short-term drug release matrix post-surgery. Importantly, the hydrogel demonstrated antimicrobial activity, particularly against *S. mutans*, the most abundant bacteria in the oral cavity associated with caries. This indicates potential use as a cavity liner, periodontal dressing, or coating for dental implants to reduce infection risk.

The primary aim of those hydrogels was to provide an antibiotic effect, which parallels the goal of our green tea hydrogel. While chlorhexidine and conventional antibiotics are very effective, a natural extract-based hydrogel might offer a gentler alternative with less risk of resistance development and additional benefits (e.g., anti-inflammatory effects from catechins).

Our antimicrobial assays did show that *E. coli* (a *Gram*–negative) was a bit less inhibited on average than *S. aureus* (*Gram*–positive), which is common since catechins often have slightly reduced efficacy on *Gram*–negative bacteria due to their outer membrane barrier. Nevertheless, the inhibition was still substantial (over 70%). *S. aureus* (Gram-positive) responded very well to the green tea hydrogel, which is promising since *S. aureus* is a common pathogen in wound infections.

Overall, based on the results obtained, we infer that the gelatin/PVA hydrogel functionalized with roasted green tea extract possesses a beneficial combination of properties: antimicrobial efficacy (especially in the oral context), appropriate degradation rate for short-term applications, and low cytotoxicity (with no evidence of promoting or inhibiting cell proliferation significantly, just maintaining viability). These characteristics position it as a potential post-surgical dressing or a vehicle for localized therapy in dentistry (for example, after periodontal surgery or tooth extraction, to prevent infection and possibly aid healing).

## 3. Conclusions

The results of this study demonstrate that a G/PVA hydrogel functionalized with roasted green tea (*Camellia sinensis*) extract exhibits antimicrobial properties, maintains cell viability, and has a favorable degradation profile. The incorporation of AERGT influenced several aspects of the hydrogel, it contributed to antioxidant and antimicrobial properties, enhancing the hydrogel’s potential for biomedical applications. Additionally, the extract may interact with gelatin and PVA via hydrogen bonding, slightly modulating the network structure, swelling behavior, and degradation profile. These findings suggest potential for future use in the dental field, specifically as a post-surgical therapeutic material or therapeutic adjunct. It provides antimicrobial activity (notably against *S. mutans*, a key oral pathogen) and degrades in a timely manner, while being soft, elastic, and non-toxic to mammalian cells. Future work on this project will explore optimizing the hydrogel’s formulation (e.g., cross-linking density to adjust degradation time) and evaluating its in vivo performance in relevant oral models. Overall, if these developments continue, this green tea-functionalized hydrogel could become a useful post-surgical treatment option, potentially replacing or reducing the need for conventional antibiotic therapies in certain clinical scenarios.

## 4. Materials and Methods

### 4.1. Materials and Equipment

The following laboratory equipment was used in this study: IKA C-MAG HS7 magnetic stirrer (IKA Works, Staufen, Germany); Thermo Scientific CL10 centrifuge (Thermo Fisher Scientific, Waltham, MA, USA); Thermo Scientific pH meter (Thermo Fisher Scientific, Waltham, MA, USA); Thermo Scientific Multiskan GO UV–Vis microplate spectrophotometer (Thermo Fisher Scientific, Vantaa, Finland); Denver Instrument analytical balance (Denver Instrument, Bohemia, NY, USA); Genie 2 Daigger vortex mixer (Daigger Scientific, Vernon Hills, IL, USA); Grant-bio McFarland densitometer (Grant Instruments, Cambridge, UK); Thermo Scientific MAXQ 6000 shaking incubator (Thermo Fisher Scientific, Waltham, MA, USA); Thermo Scientific MIDI 40 humidified incubator (Thermo Fisher Scientific, Waltham, MA, USA); MMM Incucell bacterial incubator (MMM Group, Planá, Czech Republic); Tuttnauer 2340M autoclave (Tuttnauer, Breda, The Netherlands); Thermo Scientific 1300 Series A2 biosafety cabinet (Thermo Fisher Scientific, Waltham, MA, USA); Lumistell LH–120 horizontal laminar flow hood (Lumistell, Taipei, Taiwan); Zeiss AxioCam MRc/Leica DM IL LED inverted optical microscope (Carl Zeiss Microscopy GmbH, Jena, Germany; Leica Microsystems, Wetzlar, Germany); Millipore vacuum filtration system (Merck Millipore, Burlington, MA, USA); 1000 μL, 500 μL, and 100 μL Thermo Scientific micropipettes (Thermo Fisher Scientific, Waltham, MA, USA); glass beakers and Erlenmeyer flasks (Corning PYREX, Corning Inc., Corning, NY, USA); McFarland turbidity standard tubes (bioMérieux, Marcy-l’Étoile, France).

Green tea leaves (*Camellia sinensis*, Matcha Kaori, Kyoto, Japan); type A gelatin from porcine skin (Sigma-Aldrich, St. Louis, MO, USA); poly(vinyl alcohol) (87–90% hydrolyzed, average Mw 30,000–70,000; Sigma-Aldrich, St. Louis, MO, USA); acetic acid 99% (Sigma-Aldrich, St. Louis, MO, USA); deionized water and distilled water (Karal, León, Guanajuato, Mexico); trypsin–EDTA 1% (Sigma-Aldrich, St. Louis, MO, USA); Mueller Hinton agar (BD Bioxon, Cuautitlán Izcalli, Mexico); Mueller Hinton broth (Sigma-Aldrich, St. Louis, MO, USA); chlorhexidine (CHX) 0.2% solution (FGM, Joinville, Santa Catarina, Brazil); sodium chloride (NaCl, Sigma-Aldrich, St. Louis, MO, USA); bacterial strains of *Streptococcus mutans*, *Staphylococcus aureus*, and *Escherichia coli* (ATCC, Manassas, VA, USA); human periodontal ligament stem cells (PDLSCs) primary culture; phosphate-buffered saline (PBS) tablets (BioBasic Inc., Markham, ON, Canada); MTT reagent (Thiazolyl Blue Tetrazolium Bromide, Sigma-Aldrich, St. Louis, MO, USA); collagenase type II (Worthington Biochemical Corporation, Lakewood, NJ, USA); disposable 6 mm biopsy punch (Integra Miltex, York, PA, USA); Durapore PVDF 0.22 μm filter paper (Merck Millipore, Burlington, MA, USA); Whatman #201 filter papers (Cytiva, Marlborough, MA, USA); sterile Petri dishes (Corning Inc., Corning, NY, USA); 24-well and 96-well culture plates (Corning Costar, Corning, NY, USA); 10 mL and 50 mL Falcon tubes (Corning Inc., Corning, NY, USA); 1.5 mL Eppendorf tubes (Eppendorf AG, Hamburg, Germany); sterile pipette tips (Axygen, Union City, CA, USA); inoculating loops (BD, Franklin Lakes, NJ, USA); and Parafilm sealing film (Bemis Company, Neenah, WI, USA).

### 4.2. Preparation Aqueous Extract of Roasted Green Tea (AERGT 10% w/v)

An aqueous extract of roasted green tea was prepared at 10% (*w*/*v*). Briefly, 200 mL of deionized water was heated to boil in a beaker, and 20 g of RGT leaves were added. The mixture was kept under constant stirring for 10 min and then allowed to cool. The extract was first filtered through paper, then centrifuged at 6500 rpm for 20 min and filtered again. A final vacuum filtration was performed using 0.22 μm Durapore filters to ensure clarity. The pH of the resulting green tea extract was adjusted to 10.5 using a pH meter and buffer solutions. The extract was aliquoted into sterile 15 mL Falcon tubes and stored at –20 °C until use. This extraction condition (10% *w*/*v*, 10 min) was optimized in our laboratory and has been successfully applied in our previous studies [14].

### 4.3. Preparation of Polymer Solutions

PVA Solution (7.5% *w*/*v*): 30 mL of deionized water was heated to 80 °C in a beaker. Then, 2.25 g of PVA (87–90% hydrolyzed, 30 k–70 k) was slowly added. The mixture was covered and stirred at 80 °C for 3 h until the PVA fully dissolved, yielding a clear 7.5% PVA solution.

Gelatin Solution (20% *w*/*v*): To prepare a 20% gelatin solution functionalized with roasted green tea, 6 mL of the 10% roasted green tea extract prepared above was combined with 24 mL of deionized water in a beaker. The mixture was heated to 40 °C, then 6 g of type A gelatin was added. The solution was stirred at 40 °C until the gelatin completely dissolved and the mixture became homogeneous. For the control (non–functionalized gelatin solution), the same procedure was followed but using 30 mL of water without roasted green tea extract.

### 4.4. Synthesis of Gelatin/PVA/Roasted Green Tea Hydrogel

Equal volumes of the hot 20% gelatin solution (with or without roasted green tea extract) and the hot 7.5% PVA solution were mixed (1:1 *v*/*v*) in a beaker under constant stirring at ~40 °C. The combined solution (gelatin + PVA, with or without roasted green tea extract) was stirred for 5 min to ensure complete homogenization. The resulting hydrogel solution was then poured into sterile Petri dishes (20 mL per dish), sealed with Parafilm, and refrigerated at 4 °C to gel. The formed hydrogels were stored at 4 °C until further use.

**Figure 8:**
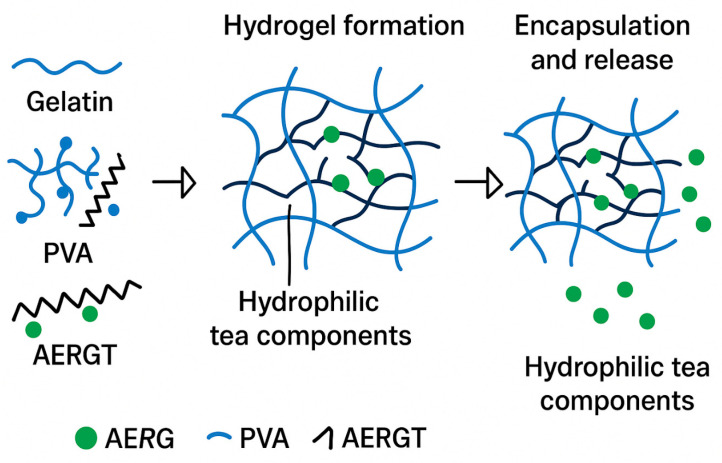
Schematic illustration of hydrogel formation, encapsulation, and release process. Gelatin, PVA, and AERGT interact to form a three-dimensional hydrogel network capable of entrapping hydrophilic tea components, which are subsequently released in a controlled manner.

Two hydrogel formulations were obtained: (1) G/PVA (gelatin/PVA hydrogel without roasted green tea, negative control) and (2) G/AERGT/PVA (gelatin/PVA hydrogel with aqueous extract of roasted green tea (Figure 8), where AE = Aqueous Extract and RGT = Roasted Green Tea). The final gelatin/PVA hydrogel incorporated AERGT at a concentration of 1% *w*/*v*. This was achieved by mixing equal volumes of the 20% gelatin solution functionalized with 2% (*w*/*v*) AERGT and the 7.5% PVA solution, resulting in a homogeneous hydrogel.

### 4.5. Primary Cell Culture

Primary human PDLSCs were isolated from periodontal ligament tissue obtained during a third molar extraction from a 21–year–old patient who provided written informed consent. The protocol was approved by the Internal Bioethics Committee of the ENES León, UNAM (registration no. CE_16/004_SN). All procedures were performed under sterile conditions in a laminar flow hood (Lumistell^®^, Celaya, Guanajuato, Mexico).

Periodontal ligament tissue explants (~1 × 1 mm) were dissected with a No. 15 scalpel blade on sterile glass slides and inoculated into 10 cm culture dishes. Explants were cultured in Minimum Essential Medium (MEM, Eagle’s formulation) supplemented with 20% fetal bovine serum (FBS), 1% antibiotics (10,000 IU/mL penicillin G and 10,000 μg/mL streptomycin; Sigma–Aldrich, Mexico, Mexico), and 1% GlutaMAX™ (Gibco, Thermo Fisher Scientific, Waltham, MA, USA). Cultures were incubated at 37 °C with 5% CO_2_ and 95% relative humidity (Thermo Fisher Scientific™) for three weeks until ~80% confluence was achieved. Medium was refreshed every three days. Subcultures were performed by washing cells twice with phosphate–buffered saline (PBS, 2 mL), followed by 1 mL of trypsin–0.05% EDTA-2Na solution, and incubating until detachment.

At passage 4, PDLSCs were seeded onto electrocharged glass slides and expanded to 90% confluence. Samples were subsequently processed at the Oral and Maxillofacial Pathology Laboratory, ENES Leon, for immunohistochemical characterization based on Kokko’s technique. Vimentin expression was detected using an optimized protocol (BioSB, Santa Barbara, CA, USA), collagen deposition was assessed by Masson’s trichrome staining (Hycel, Zapopan, Jalisco, Mexico) and negative for CD34 and CD56 (Sigma–Aldrich, Burlington, MA, USA). Cells were examined with a Leica DM750 optical microscope (Leica Microsystems, Wetzlar, Germany) at 40× magnification.

### 4.6. Cytotoxicity Assay

Cytotoxicity was evaluated in accordance with ISO 10993-5:2009 [15]. Human periodontal ligament stem cells (PDLSCs) were sub cultured in Minimum Essential Medium (MEM; Sigma-Aldrich, St. Louis, MO, USA) supplemented with 10% fetal bovine serum (FBS; Sigma-Aldrich), penicillin–streptomycin (10,000 IU/mL and 100 mg/mL, respectively; Sigma-Aldrich), and 1% GlutaMAX (Gibco, Grand Island, NE, USA). Cultures were maintained at 37 °C in a humidified incubator with 5% CO_2_.

PDLSCs were cultured in standard growth medium and, at ~80% confluence, were trypsinized for seeding into a 24–well plate. Approximately 3 × 10^5^ cells/mL were seeded per well (300 μL per well of growth medium). Six wells were used for cell–only control, three wells for the G/PVA hydrogel, and three wells for the G/AERGT/PVA hydrogel (experimental group).

After 24 h of cell attachment, the medium was removed from all wells. Both hydrogel samples (G/PVA and G/AERGT/PVA) were cut with a 6 mm diameter biopsy punch and then halved to yield ~6 mm × 3 mm hydrogel pieces. One piece of hydrogel was placed in each designated well (three wells for each hydrogel group). The six control wells contained cells with no hydrogel. The plate was incubated for 24 h at 37 °C in a 5% CO_2_ humidified atmosphere.

Following the 24 h incubation with hydrogels, the medium was aspirated from all wells. The experimental wells were gently washed with 300 μL of PBS (to remove any extracts or debris) and aspirated; this wash was performed twice. Then, 300 μL of MTT solution (0.5 mg/mL in culture medium) was added to each well (controls and hydrogel–exposed cells) and the plate was incubated for another 24 h at 37 °C. After this period, the MTT solution was removed, and 300 μL of DMSO was added to each well to dissolve the formazan crystals. Finally, 100 μL from each well was transferred to a 96–well plate (with 6 technical replicates per group) for reading. The absorbance of each sample was measured at 595 nm using a UV–Vis microplate spectrophotometer. Cell viability (%) was calculated relative to the untreated control (PDLSCs with no hydrogel). Each condition was tested in triplicate (three independent experiments, n = 9 total). The results were averaged and used for comparative analysis of viability.

### 4.7. Enzymatic and Hydrolytic Degradation Tests

Hydrogel degradation was evaluated under three conditions: hydrolytic (in PBS), enzymatic with trypsin (a protease), and enzymatic with collagenase II. Hydrogel discs (~6 mm diameter) of G/PVA and G/AERGT/PVA were prepared using a biopsy punch. For each condition, initial weights of the hydrogel samples were recorded (at t = 0). The samples were then placed in 1.5 mL Eppendorf tubes containing 1 mL of either PBS (pH 7.4), trypsin–EDTA solution (1%), or collagenase II solution (prepared according to manufacturer guidelines). The tubes were incubated at 37 °C in a shaking incubator at 60 rpm.

Weight loss over time was measured by stopping the shaking every 10 min, gently blotting and weighing each hydrogel sample, and then returning it to the solution. This process was repeated at 10 min intervals for up to 80 min (or until the hydrogel completely degraded/disintegrated). At each 10 min interval, the supernatant from each tube was saved for absorbance measurement (to monitor solubilized components as an indirect measure of degradation).

The collected supernatants from each time point (t = 10, 20, 30, 40, 50, 60, 70, 80 min) were analyzed by measuring absorbance at 350 nm (which is indicative of solubilized peptide fragments or tea polyphenols) using a 96-well plate in the UV-Vis spectrophotometer. Separate sets of experiments were conducted for the two enzyme conditions (trypsin and collagenase) and the hydrolytic condition (PBS alone). All tests were performed in triplicate.

### 4.8. Antibacterial Activity: Agar Diffusion Assay

The antimicrobial effect of the hydrogels was first assessed by an agar diffusion (inhibition zone) test. Bacterial cultures of *S. mutans*, *S. aureus*, and *E. coli* grew to the 0.5 McFarland standard (1 × 10^8^ CFU/mL). A working suspension for each bacterium was prepared by diluting the culture in 0.85% NaCl solution (6 mL NaCl + 1 mL bacterial culture).

Mueller Hinton agar plates were prepared (three plates for each bacterial species). On each agar plate, the bacterial suspension was spread uniformly across the surface using a sterile swab (the lawn culture method). Five treatment groups were tested on each plate: (1) G/AERGT/PVA hydrogel, (2) G/PVA hydrogel, (3) positive control (CHX 0.2% solution), (4) negative control (0.85% NaCl solution), and (5) blank paper disk. Two 6 mm wells were punched into the agar for placing the hydrogel samples (one well for G/AERGT/PVA and one for G/PVA). Hydrogel discs (6 mm) were cut and placed into these wells. Sterile filter paper disks were used for the controls: a paper disk soaked with 5 μL of 0.2% chlorhexidine solution served as the positive control, a disk soaked with 5 μL of 0.85% NaCl served as a solvent negative control, and a plain disk with no addition served to check for any inherent effect of the paper. All five samples (two hydrogels and three controls) were placed equidistantly on each agar plate.

The plates were sealed with Parafilm and incubated at 37 °C for 24 h. After incubation, each plate was examined for zones of inhibition (clear halos) around the sample placements. The diameter of the inhibition halo (in millimeters) was measured for each sample in each plate. Three independent diffusion assays were performed for each bacterial species (n = 3, three plates per species).

### 4.9. Antibacterial Activity: Microdilution Assay

A microdilution assay was conducted to quantitatively assess bacterial growth inhibition by extracts from the hydrogels. Fresh 0.5 McFarland bacterial suspensions were prepared for *S. mutans*, *S. aureus*, and *E. coli* as described above. For each bacterial strain, 10 mL of Mueller Hinton broth was inoculated with 100 μL of the standardized bacterial suspension in a sterile 15 mL Falcon tube (preparing a working inoculum).

Sterile 24-well plates were used for the assay, with one plate per bacterial species. Hydrogel discs (6 mm) of G/AERGT/PVA and G/PVA were cut and then cut in half (to increase surface area relative to volume). Half-disc samples of each hydrogel were placed into designated wells (we tested three wells for each hydrogel type). For the positive control, 100 μL of the working inoculum was mixed with 100 μL of 0.12% chlorhexidine in a well (three wells for each species). For the negative control, 100 μL of the working inoculum without any hydrogel or chlorhexidine was used (these untreated wells represent 100% growth). Each well was then filled up to 1 mL total volume with Mueller Hinton broth as needed. Thus, for each bacterium we had wells with G/AERGT/PVA hydrogel + bacteria, wells with G/PVA hydrogel + bacteria, positive control wells with CHX + bacteria, and untreated control wells with bacteria only. All wells were covered and incubated at 37 °C for 24 h.

After 24 h, the content of each well was mixed and 100 μL was transferred to a new 96–well plate. To each of these 100 μL samples, 10 μL of MTT solution (5 mg/mL in PBS) was added. The 96–well plate was incubated for 3 h at 37 °C. Then, the wells were gently aspirated and 100 μL of DMSO was added to each to dissolve formazan. After 1 h incubation with DMSO, the absorbance at 595 nm was measured for each well using the microplate reader. The percentage of bacterial viability (or conversely, inhibition) was calculated by comparing the absorbance of each treatment well to the untreated control (100% growth). Three independent experiments were performed for each bacterial strain (with triplicate wells per condition each time, n = 9).

### 4.10. Evaluation of the Hydrogel for Osteoblastic Differentiation

To investigate the osteogenic potential of the G/AERGT/PVA hydrogel, periodontal ligament stem cells (PDLSCs) were cultured under controlled differentiation conditions. Cells were seeded in osteogenic medium consisting of minimum essential medium (MEM) supplemented with 10% fetal bovine serum (FBS), 1% antibiotics, 0.1 mM dexamethasone, 5 mM β-glycerophosphate, 50 µg/mL ascorbic acid, 20 ng/mL transforming growth factor-β3, and 5 ng/mL fibroblast growth factor–2. Parallel cultures of standard subpassage cells served as negative controls. The hydrogel constructs were incubated with PDLSCs for ten consecutive days to allow matrix deposition and induction of differentiation. Mineralization was subsequently assessed by Alizarin Red staining, a classical method for detecting calcium phosphate deposits within the extracellular matrix. After staining, bound dye was solubilized in a solution of 5% 2–isopropanol and 10% acetic acid for 16 h. Quantification of mineral deposition was performed by spectrophotometric analysis at 550 nm, providing a direct measure of calcified nodule formation and validating the osteoinductive effect of the G/AERGT/PVA hydrogel.

## Figures and Tables

**Table 1:** Bacterial inhibition zones (mm).

Assay	Groups	Trial 1	Trial 2	Trial 3	Mean	SD
*S. mutans*	G/AERGT/PVA	7.75	9.5	8.25	8.5	0.901387819
	CHX 0.12%	10.75	10	9.75	10.17	0.5204165
	G/PVA	4.75	5.25	4.25	4.75	0.5
*S. aureus*	G/AERGT/PVA	6.5	7.5	7.75	7.25	0.661437828
	CHX 0.12%	9	9	9.25	9.083	0.144337567
	G/PVA	4	4	5	4.333	0.577350269
*E. coli*	G/AERGT/PVA	7.75	6.5	7.5	7.25	0.661437828
	CHX 0.12%	8	8.25	8.5	8.25	0.25
	G/PVA	7.25	5	7.5	6.583	1.376892637

Statistical analysis was performed using non-parametric test by the Kruskal–Wallis followed by Mann–Whitney multiple comparisons. No statistically significant differences were observed between the groups (*p* > 0.05, n = 3).

## Data Availability

The data presented in this study are available on request from the corresponding authors.
